# DEP-On-Go for Simultaneous Sensing of Multiple Heavy Metals Pollutants in Environmental Samples

**DOI:** 10.3390/s17010045

**Published:** 2016-12-27

**Authors:** Madhu Biyani, Radhika Biyani, Tomoko Tsuchihashi, Yuzuru Takamura, Hiromi Ushijima, Eiichi Tamiya, Manish Biyani

**Affiliations:** 1 BioDevice Technology Ltd., 2-13 Asahidai, Nomi City, Ishikawa 923-1211, Japan; drmadhubiyani@gmail.com (M.B.); tsuchihashi@biodevicetech.com (T.T.); ushijima@biodevicetech.com (H.U.); tamiya@ap.eng.osaka-u.ac.jp (E.T.); 2 Biyani BioSolutions Pvt. Ltd., Biyani Research Group, R-4, Sector 3, Vidhyadhar Nagar, Jaipur 302023, India; biyaniradhika@yahoo.in; 3 Department of Bioscience and Biotechnology, Japan Advanced Institute of Science and Technology, 1-1 Asahidai, Nomi City, Ishikawa 923-1292, Japan; takamura@jaist.ac.jp; 4 Department of Applied Physics, Graduate School of Engineering, Osaka University, 2-1 Yamadaoka, Suita, Osaka 565-0871, Japan

**Keywords:** heavy metal ions, environmental analysis, electrochemical sensor, screen printed electrode palm-sized potentiostat

## Abstract

We describe a simple and affordable “Disposable electrode printed (DEP)-On-Go” sensing platform for the rapid on-site monitoring of trace heavy metal pollutants in environmental samples for early warning by developing a mobile electrochemical device composed of palm-sized potentiostat and disposable unmodified screen-printed electrode chips. We present the analytical performance of our device for the sensitive detection of major heavy metal ions, namely, mercury, cadmium, lead, arsenic, zinc, and copper with detection limits of 1.5, 2.6, 4.0, 5.0, 14.4, and, 15.5 μg·L^−1^, respectively. Importantly, the utility of this device is extended to detect multiple heavy metals simultaneously with well-defined voltammograms and similar sensitivity. Finally, “DEP-On-Go” was successfully applied to detect heavy metals in real environmental samples from groundwater, tap water, house dust, soil, and industry-processed rice and noodle foods. We evaluated the efficiency of this system with a linear correlation through inductively coupled plasma mass spectrometry, and the results suggested that this system can be reliable for on-site screening purposes. On-field applications using real samples of groundwater for drinking in the northern parts of India support the easy-to-detect, low-cost (<1 USD), rapid (within 5 min), and reliable detection limit (ppb levels) performance of our device for the on-site detection and monitoring of multiple heavy metals in resource-limited settings.

## 1. Introduction

Heavy metal-induced toxicity is an uncommon issue in medical diagnostics and can result in significant morbidity and mortality. There are more than 20 heavy metals [1], some of which, such as zinc (Zn) and copper (Cu), are essential for good health in trace amounts. However, excessive exposure to these heavy metals can cause serious health hazards in living organisms because they are not metabolized by the body and, thus, they accumulate in the soft tissues and are associated with negative effects on a series of metabolic pathways [2,3]. Some typical heavy metals, such as lead (Pb), cadmium (Cd), arsenic (As), and mercury (Hg), are prime examples that are considered systemic toxicants to the human body, even at low levels of exposure. These metals can induce multiple organ damage, including neurological impairment, autoimmune diseases, joint disease, kidney and nervous system damage [4,5,6,7]. In addition, several reports based on epidemiological and experimental studies have shown that long-term sub-threshold exposure to these metals results in the promotion of carcinogenesis [8,9,10]. These heavy metals generally enter our environment by natural means, such as soil erosion, geological weathering, and anthropogenic activities, such as industrial effluents, urban runoff, and sewage discharge. Since the contribution of these sources can vary both spatially and seasonally, information on these variations is crucial. The regular monitoring and detection of heavy metal pollutants is a serious and urgent concern for global public health. Furthermore, the synergistic toxicity of multiple heavy metals on human liver cells has recently been reported, and the results suggested that combined effects should be considered in risk assessments of heavy metal-induced toxicity [11]. Therefore, the simultaneous detection of multiple heavy metals is also of paramount importance to human health. The next challenge is to enable heavy metal testing to be performed in the resource-limited settings that accounts for more than 80% of the world’s population.

Technological advances in analytical chemistry have presented a plethora of sensor systems. However, a real-time and on-field detection system for the continuous environmental monitoring of toxic heavy metal pollutants is yet not fully realized. Traditional quantitative methods, such as inductively-coupled plasma atomic emission spectroscopy (ICP-AES), inductively-coupled plasma mass spectrometry (ICP-MS), and graphite furnace atomic absorption spectrometry (GFAAS) [12], require a large set-up, high cost, and sophisticated instrument-equipped laboratories for sample analysis. These methods, therefore, cannot be applied to develop real-time and on-site monitoring devices with characteristic features of portability, affordability, and simplicity. Since most heavy metals are colorless and odorless, the chances of being unwittingly exposed to them are high. To protect human health, as well as to engage in the rapid on-site monitoring of heavy metal pollutants, a portable handheld device for detecting multiple heavy metals below the permissible range in the exposure samples should be prioritized.

Electrochemistry offers a simple alternative to traditional analytical methods and provides a high degree of sensitivity, and it has been shown to be highly effective for device miniaturization. The introduction of screen-printing electrode technology by our group [13,14,15] (called a disposable electrode printed (DEP)-chip) and other groups [16,17,18] has further widened the scope of available electrochemical sensors for large-scale applications. Furthermore, the recent development of a portable and low-cost potentiostat by our group (MiniSTAT, BioDevice Tech, Ishikawa, Japan) and others (CheapSTAT) showed the advantages of electrochemical detection in a POC setting. Very recently, the Whitesides group coupled an electrochemical method with telecommunications technology for resource-limited applications [19]. In this paper, we developed a ready-to-use, simple, easy-to-use, inexpensive, mobile electrochemical sensing device for monitoring of multiple heavy metals using our originally-developed DEP-chip and palm-sized potentiostat device. We evaluated and showed the system’s versatility for the sensitive, selective and simultaneous determination of heavy metal ions in real samples from groundwater for drinking, tap water, environmental dust, soil and industry-processed solid foods samples, including rice and noodles. We also showed the on-field applications of our device for the rapid on-site detection of heavy metals in drinking groundwater in some contaminated areas in the northern parts of India.

## 2. Materials and Methods

### 2.1. Materials and Apparatus

Stock standard solutions (1000 mg·L^−1^) of cadmium, lead, arsenic, zinc, and copper were purchased from Nacalai Tesque, Inc. (Kyoto, Japan). The supporting electrolytes used in the experiments were HCl (0.1 or 1.0 M) or acetate (0.2 M, pH 4.6) buffer solutions with bismuth nitrate pentahydrate (2 mg·L^−1^) (Kanto Chemical Co. Inc., Tokyo, Japan). Deionized water (not less than 18.2 MW·cm^−1^), which was obtained from RFD240NA, ADVANTEC (Toyo Roshi Company Ltd., Chiba, Japan), was used throughout the experiments. The tap water samples were obtained from our laboratories in India and Japan. The rice and noodle samples were purchased from supermarkets in India and Japan. The indoor dust samples were collected from several rooms in two buildings in India and Japan.

The disposable electrode-printed chip (DEP chip) was provided by Bio Device Technology Co. Ltd. (Ishikawa, Japan). The DEP chip consisted of a miniaturized three-electrode system including a carbon or gold working electrode, a carbon counter electrode and an Ag/AgCl reference electrode. The total length of the DEP chip was 12 mm, and the area of the working electrode was 2.64 mm^2^. Differential pulse voltammetery (DPV) measurements were taken with a Potentiostat BDT miniSTAT 100 (Bio Device Technology Co. Ltd.) connected to a Windows tablet (Acer One) that was loaded with electrochemical system software (KME USBstat version 2, Bio Device Technology Co. Ltd.) All measurements were performed at room temperature (25 °C). For the DPV measurements, each DEP chip was used once and discarded after each measurement to avoid contamination during analysis.

### 2.2. Preparation of Standard Solutions

The standard solutions of cadmium, lead, arsenic, zinc, mercury, and copper were prepared from the standard stock by serial dilutions. The calibration solutions of cadmium, lead, zinc, mercury, and copper were diluted in 0.2 M acetate buffer solution (pH 4.6) and arsenic and mercury were prepared in 1 M and 0.1 M HCl solutions, respectively. However, for simultaneous detection of As and Hg, mixture was prepared in 0.1 M HCl, 0.5 M NaCl supporting buffer. A screen-printed carbon electrode has been tested with negligible effect of oxygen on the background signal in comparison to glassy carbon electrodes, so the electrolyte solution in this study was used without removing oxygen from the sample [20].

### 2.3. Pre-Treatments of Real Samples

Tap water, rice, noodles, dust, and soil samples were selected for use as real samples. All these samples were pretreated by a fast pretreatment process for on-field determination of heavy metals in environmental samples. Tap water was collected in clean bottles after rinsing the bottle 3–5 times with tap water, followed by adjusting the pH to 4.6 with 0.1 M acetic acid and 4 M NaOH solution. The collected tap water was then mixed with 0.2 M acetate buffer (pH 4.6) in ratio of (1:1). The standard addition method was applied to pre-treated tap water for simultaneous detection of Zn, Cd, Pb, Cu, As, and Hg. For this, tap water samples were prepared by mixing 200 μL of pre-treated tap water with 200 μL of standard mixture (mixture-I is Zn:Cd:Pb:Cu:Hg 150:5:5:25:25 μg·L^−1^, mixture-II is Zn:Cd:Pb:Cu:Hg 300:10:10:50:50 μg·L^−1^, and mixture-III is Zn:Cd:Pb:Cu:Hg 450:20:20:75:75 μg·L^−1^) prepared in a reagent solution containing 2 mg∙L^−1^ of bismuth nitrate pentahydrate in 0.2 M acetate buffer (pH 4.6). For determination of As and Hg, tap water samples were prepared by adding 200 μL pre-treated tap water with three consecutive standard mixtures (mixture-I As:Hg 10:10 μg·L^−1^, mixture-II As:Hg 30:30 μg·L^−1^; and mixture-III As:Hg 60:60 μg·L^−1^) prepared in a supporting electrolytes solution (0.1 M HCl, 0.5 M NaCl solution).

For rice and noodle samples, 1 g finely ground powder was added to 10 mL of 0.1 M acetic acid solution and allowing it to stand for 1 h while agitating with a magnetic stirrer. The mixture was then filtered (using Whatsman filter paper 42 no. and 0.45 μm filtered membrane) and pH was adjusted to 4.6 with 0.1 M acetic acid and 4 M NaOH solution. The solutions were then mixed with 0.2 M acetate buffer (pH 4.6) in a ratio of 1:4. For the quantity measurement of Cd in rice and Pb in noodle, the standard addition method was applied. The rice samples were prepared by adding 200 μL rice samples with 200 μL of Cd (20/40/60 μg·L^−1^) standard solutions and noodle samples were prepared by adding 200 μL noodle sample with Pb (30/60/90 μg·L^−1^) prepared in a reagent solution containing 2 mg·L^−1^ bismuth in 0.2 M acetate buffer (4.6).

The dust samples were prepared by collecting fresh indoor dust from several rooms in two buildings (Jaipur city in India and Nomi City in Japan) with the aid of a vacuum cleaner. The dust samples were filtered to remove any visible impurities, e.g., hair, paper and other garbage, and then 1 g was added to 10 mL of 0.2 M acetic acid solution and allowing it to stand 1 h while stirring with magnetic stirrer. The mixture was then centrifuged to collect the aqueous phase. The pH of aqueous phase was adjusted to 4.6 with 4 M NaOH solution. The collected aqueous phase was mixed with 0.2 M acetate buffer (pH 4.6) in the ratio of 1:4. For simultaneous determination of Zn, Cd, Pb, Cu, As, and Hg in dust samples, the standard addition method was applied. The samples were prepared by mixing 200 μL of dust sample with 200 μL of standard mixture as indicated earlier (mixture-I is Zn:Cd:Pb:Cu:Hg 100:50:100:20:50 μg·L^−1^; mixture-II is Zn:Cd:Pb:Cu:Hg 300:150:300:60:150 μg·L^−1^; mixture-III Zn:Cd:Pb:Cu:Hg 400:200:400:80:200 μg·L^−1^) prepared in a reagent solution containing 2 mg·L^−1^ of bismuth nitrate pentahydrate in 0.2 M acetate buffer (pH 4.6). As and Hg in dust samples were also determined by standard addition method (mixture-I As:Hg 50:25 μg·L^−1^; mixture-II As:Hg 100:50 μg·L^−1^; mixture-III As:Hg 200:100 μg·L^−1^; mixture-IV As:Hg 300:200 μg·L^−1^) in a supportive solution (0.1 M HCl, 0.5 M NaCl).

The soil sample was taken from 0 cm to 10 cm depth from the garden in Nomi City, Japan. The soil sample was air dried to constant weight and passed through 1 mm sieve. Then 1 g of dried soil was diluted to 10 mL of 0.1 M acetic acid. After agitating for one hour, the soil sample was centrifuged to separate the aqueous phase from solid phase. The pH of collected aqueous phase was adjusted to pH 4.6 with 4 M NaOH solution and diluted to 0.2 M acetate buffer (pH 4.6) in a ratio of 1:4. For simultaneous determination of Zn, Cd, Pb, Cu, As, and Hg in soil samples, the standard addition method was applied. The samples were prepared by mixing 200 μL of soil sample with 200 μL of standard mixture (mixture-I Zn:Cd:Pb:Cu:Hg 100:10:10:50:50 μg·L^−1^; mixture-II Zn:Cd:Pb:Cu:Hg 200:20:20:100:100 μg·L^−1^; mixture-III Zn:Cd:Pb:Cu:Hg 300:30:30:150:150 μg·L^−1^). Standard solutions of As- and Hg-added 200 μL of soil samples were prepared (mixture-I As:Hg 10:10 μg·L^−1^; mixture-II As:Hg 20:20 μg·L^−1^; mixture-III As:Hg 60:60 μg·L^−1^; mixture-IV As:Hg 90:90 μg·L^−1^) in a supporting electrolyte solution (0.1 M HCl, 0.5 M NaCl).

In our present protocol, a pre-treatment step for non-liquid samples requires additional laboratory preparations. However, this can be further simplified by solid-phase extraction method using functionalized magnetic nanoparticles. For comparative analysis, few samples, including drinking groundwater, tap water, rice, noodle, and dust were prepared for the individual determination of heavy metal with or without added standard solution of Pb, Cd, and As. All these prepared samples (50 mL) were used for ICP-MS analysis (Yamato Kankyo Bunseki Centre, Ishikawa, Japan) and also analyzed with DEP-On-Go device.

### 2.4. Procedure for DPV Analysis

The DEP chip was attached to a miniStat100 potentiostat connector. The miniStat100 potentiostat is supplied with power via a USB-connected tablet. A 30-μL volume of pre-treated sample solution was dropped onto the DEP chip for measurement. The heavy metal detections were performed by differential pulse voltammetry (DPV) after deposition of heavy metals using a potentiostat (miniStat100) run by KME software with optimized DPV parameters (Table S1). The parameters E1 and T1 in Table S1 represent the deposition potential and time. We set the DPV measurement conditions (deposition potential, deposition time and scan rate) according to the stripping behavior of each metal ions on working electrode surface, and then followed by DPV scanning in a series of superimposed staircase potential waveform.

## 3. Results

### 3.1. Characteristics of the “DEP-On-Go” Sensor 

**Figure 1:**
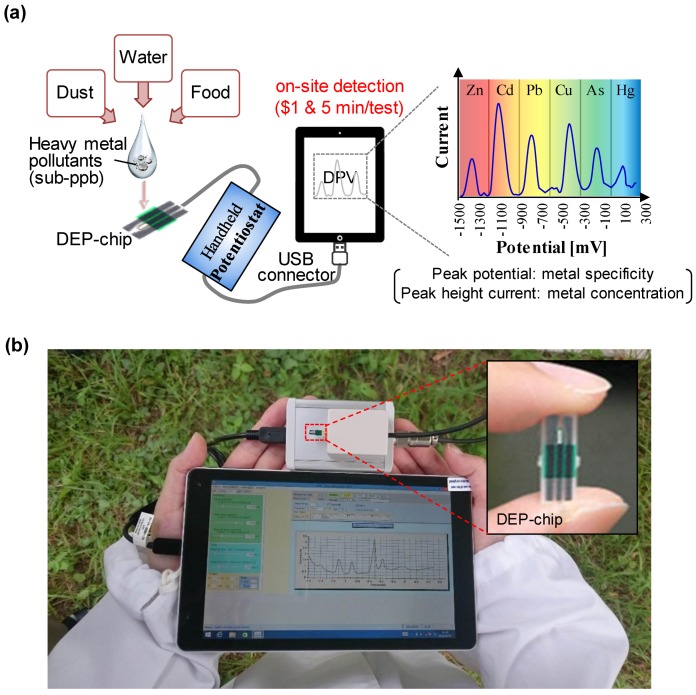
“DEP-On-Go sensing” concept. Schematic representation of the work flow (**a**) and photographs (**b**) of the sensing platform for the on-site and rapid detection of heavy metal pollutants directly from environmental samples.

Figure 1a illustrates the working flow of “DEP-On-Go” sensor that uses the anodic stripping voltammetry (ASV) technique for the rapid on-site detection of multiple heavy metal ions. The primary components of this system are originally developed and are a disposable electrode-printed chip (DEP chip), a USB-powered palm-sized potentiostat (BDT miniStat100, Bio Device Technology Co. Ltd.), and proprietary software (KME_USBStat_v2, Bio Device Technology Co. Ltd.) that was loaded on a Pocket PC. These components and other measurement tools are compactly packed into a lightweight briefcase for on-field sample-to-answer analysis. Trace amounts of heavy metals can be detected by ASV using differential scanning techniques such as differential pulse (DP) or square wave (SW) stripping steps [21]. In principle, metal ions are electrochemically reduced and oxidized on a working electrode by applying potential. This chemical reaction can then be transformed into strong electrical current peaks. The intensity of the current peak represents the concentration of heavy metals in the sample. Each metal shows a specificity to oxidize at a particular potential. Figure 1b shows a picture of the “DEP-On-Go” device, which is designed for a mobile, rapid, affordable, and reliable on-site determination of environmental samples. The corresponding video of on-site field operations can be seen in the supporting information (S1 movie). Our palm-sized potentiostat can be programmed to perform a variety of electrochemical measurements (chronoamperometry, cyclic voltammetry, differential pulse voltammetry, square wave voltammetry, and linear sweep voltammetry). We show the electrochemical capabilities of our palm-sized potentiostat by comparing a cyclic voltammograms to a bench-top potentiostat (Figure S1). The comparative features of our device with conventional technique (ICP-MS) are described in Table S2. The DEP-On-Go device can be more advantageous for on-site analysis for several reasons such as low-cost setup, affordability (the running cost per sample is less than one US dollar), ppb-level sensitivity (which can be improved further), rapid analysis (sample-to-answer in minutes), and a simple-to-use setup for minimally-trained users.

### 3.2. Standardization of the “DEP-On-Go” Sensor for Multiple Heavy Metal Detection 

**Figure 2:**
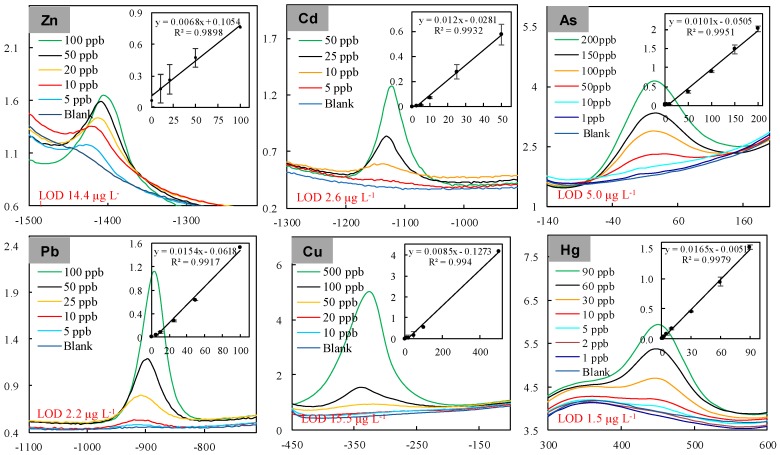
Individual electrochemical measurement of heavy metals in a standard solution using the “DEP-On-Go” system. DP voltammograms and corresponding calibration curves (inset) are shown for the detection of zinc, cadmium, lead and copper using a carbon DEP chip, and arsenic, and mercury using a gold DEP chip. The *X*-axis and *Y*-axis represent the potential (mV) and the current (μA), respectively. The *X*-axis and *Y*-axis in the insets represent the concentrations of metals (μg·L^−1^) and calculated peak current heights (μA), respectively. The data are the averages of four to six independent experiments. LOD: limit of detection.

Firstly, we evaluated the analytical performance of the “DEP-On-Go” sensor by calibrating with different concentrations of the individual heavy metal standards in differential pulse voltammetry (DPV) mode. Figure 2 shows the DP voltammograms and obtained calibration plots for detecting zinc, copper, lead, cadmium, arsenic, and mercury using unmodified, screen-printed electrodes based on carbon and/or gold DEP chips. A clear and single peak current response was observed on the carbon DEP chip for zinc, cadmium, lead, and copper standards at average potentials of approximately −1.423, −1.178, −0.889, and −0.321 V, respectively, and with detection limits of 14.4, 2.6, 4.0, and 15.5 μg·L^−1^, respectively (Table 1). Our observation that employed low stripping peaks for mercury and no peak for arsenic indicated that carbon DEP chips cannot be applied to the sensitive determinations of mercury and arsenic. Therefore, we used gold DEP chips for the sensitive detection of mercury and arsenic. Interestingly, a clear, single, and improved peak current response was observed on the gold DEP chip for arsenic and mercury at average peak potentials of approximately at 0.092 and 0.450 V, respectively, and with detection limits of 5.0, and 1.5 μg·L^−1^, respectively (Table 1). All of the calibration curves showed linearity with correlation coefficients ranging from 0.9979 to 0.9898. Based on these data, we determined the detection efficiency of our DEP chip system with acceptable sensitivity. Recently, modified electrodes, especially those with conducting polymers such as graphite-polyurethane or carbon nanotube modified electrode, have been developed to enhance selectivity and sensitivity [22,23,24]. Interestingly, our portable DEP-On-Go system could be able to detect heavy metals without any special electrode modifications and with acceptable sensitivity at ppb levels which are significantly lower (1–3 orders of magnitude) than the threshold or permissible level imposed by the environmental protection agency WHO (World Health Organization) [25]; OSHA (Occupational Safety and Health Administration of United States, Department of Labor) [26]; US-EPA (United States Environmental Protection Agency) [27] (Table 1).

**Figure 3:**
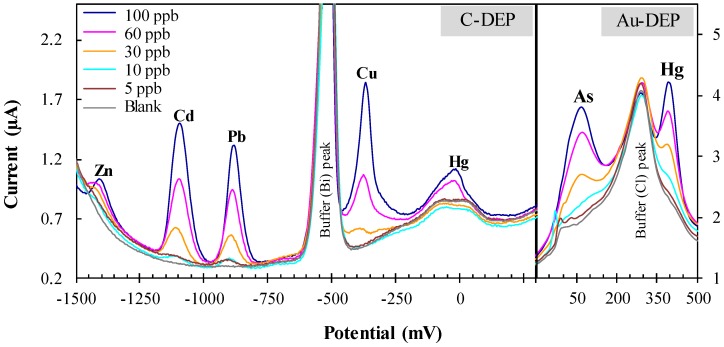
Simultaneous electrochemical measurement of six heavy metals in a standard solution using the “DEP-On-Go” system. DP voltammograms are shown for the detection of zinc, cadmium, lead, and copper using a carbon DEP chip (C-DEP), and arsenic and mercury using a gold DEP chip (Au-DEP) in standard solutions. The data are the averages of four to six independent experiments.

Next, we evaluated the performance of our system for the simultaneous electroanalytical determination of multiple heavy metal standards. The simultaneous detection of several heavy metals is advantageous over time/cost-consuming manual operations, particularly when real samples, such as drinking groundwater are polluted with a mixture of several heavy metals. Nevertheless, the signal of the target metal often experiences interference because of the presence and co-deposition of other heavy metals onto the active site of the electrode surface. Therefore, we also evaluated the performance of our system for the simultaneous electroanalytical determination of lead, cadmium, zinc, and copper using carbon DEP chips and arsenic and mercury using gold DEP chips. The application of chemically-modified carbon working electrodes has provided the simultaneous detection of heavy metals in water samples with enhanced specificity [28,29]. Bismuth is an environmentally-friendly element with a property to form “fused alloys” with heavy metals which facilitates the nucleation process during accumulation of heavy metals. In addition, bismuth has been widely used for anodic stripping voltammetry to improve stripping characteristics including well-defined and undistorted stripping signal and excellent resolution of neighboring peaks together with high sensitivity [30,31]. Therefore, to enhance the electroanalytical performance of carbon DEP chips for simultaneous detection, we optimized the operational parameters, including the addition of the bismuth directly to the sample solution and the simultaneous deposit of target metals on the carbon electrode. We simultaneously measured a standard mixture of zinc, cadmium, lead, copper, and mercury for a concentration range between 5 and 100 μg·L^−1^ with all well-defined and separated peaks (Figure 3). The obtained calibrated result displayed the addition effect on all tested metals for their peak current heights with a liner coefficient value (with correlation coefficients ranging from 0.9968 to 0.9672) and without any interference (Figure S2). This result confirms that the bismuth treatment eliminates cross-interferences, and, thus, the simultaneous determination of zinc, cadmium, lead, and copper was successfully achieved and was as sensitive as of individual determination, the exception being mercury as the limit of the detection sensitivity for mercury on the carbon DEP chip was decreased to 93.3 μg·L^−1^ (nearly 100-fold less than the sensitivity of the gold DEP chip). We then analyzed and confirmed that arsenic and mercury can be detected sensitively and simultaneously with well-defined and separated peaks using a gold DEP chip (Figure 3). One additional background peak due to supporting electrolytes solution was also observed between arsenic and mercury. These findings confirm that all studied six heavy metals can be detected simultaneously using a simple and unmodified DEP-On-Go system.

### 3.3. Simultaneous Detection of Multiple Heavy Metals in Real Environmental Samples

**Figure 4:**
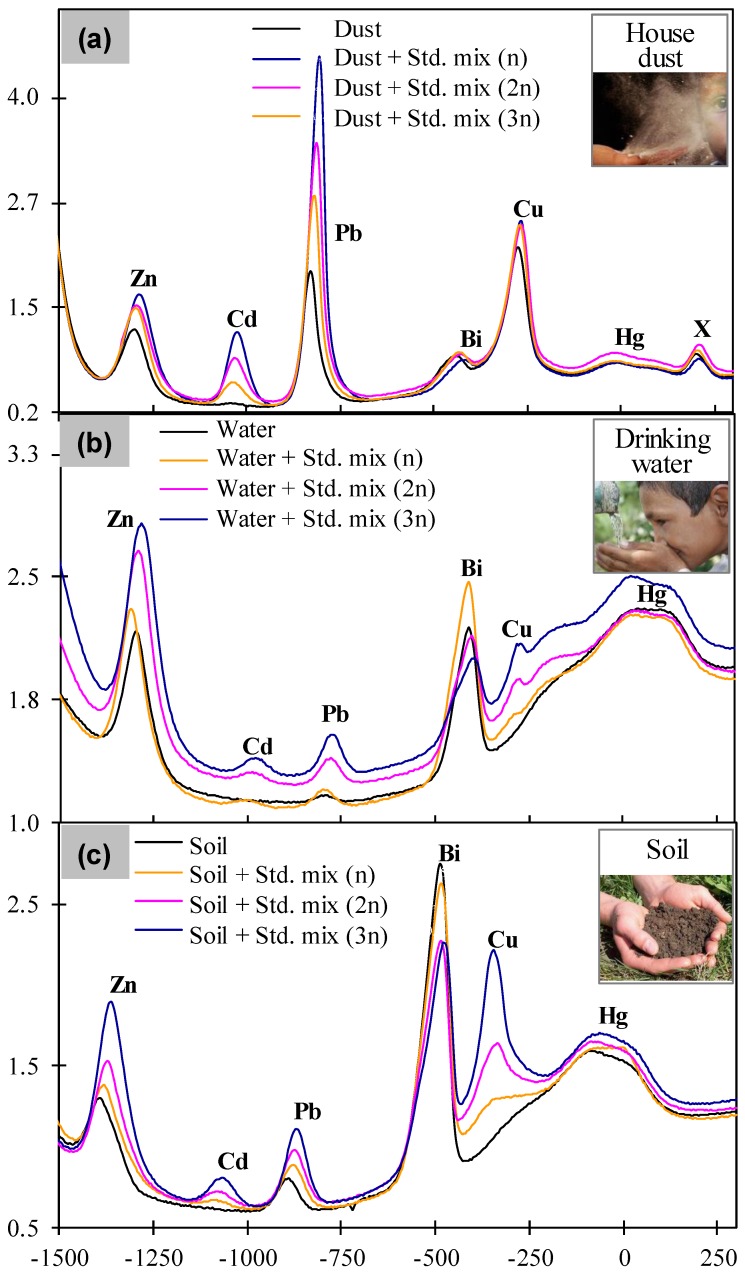
Simultaneous electrochemical measurement of various heavy metals in real samples using the “DEP-On-Go” system. DPV curves are shown for the simultaneous detection of zinc, cadmium, lead, copper, and mercury in real samples from house dust (**a**), drinking groundwater (**b**), and soil (**c**). Std. mix = Standard mixture (see Section 2.3).

With the aim of verifying the practicability of the proposed DEP-On-Go system for simultaneous monitoring, we measured the heavy metal contaminants in real environmental samples including the house dust, the groundwater for drinking, and the soil from several localities in the cities of Jaipur (India) and Nomi (Japan). A total of six or more well-defined and separated metal peaks were observed in all the three samples (Figure 4), which was further confirmed by the standard addition method, i.e., adding known concentrations of each metal in the real samples. The calibrated results (Figures S3–S5) were used to calculate the concentrations for all the metals detected in the real samples. The results of house dust showed that dust samples from the urban area in India contained high levels of lead, and possibly mercury, however, other metals were extremely lower than the maximum permissible limit set by OSHA. The World Health Organization (WHO) reported tolerable monthly intakes of Cd as 25 μg·kg^−1^ body weight and daily intakes of Pb as 3 μg kg^−1^ for all human groups [27]. Interestingly, one additional peak (X) was also observed in dust sample which may be representing chromium (Figure 4a). The appearance of several other heavy metals detected by ICP-MS in the dust sample in significantly higher amounts, such as Fe, Mn, Cr, Ni, Zr, revealed the need of routine testing of environmental dust samples for early warning. Next, the result of drinking groundwater showed that all of the detected heavy metals except copper and zinc were nearly close to the maximum permissible exposure limit set by WHO. The result of soil that was collected from a garden area showed all detected heavy metals were extremely lower than the maximum permissible limit set by US-EPA. Based on these results, and owing to the simplicity, affordability, portability, and performance, DEP-On-Go device was successfully demonstrated to monitor heavy metal toxicity in environmental samples for early warning.

Furthermore, to show the ability to perform heavy-metal tests in products from the processed food industry, we measured the cadmium concentrations in rice and lead in noodle samples that were collected from India and Japan. Very recently, food safety regulations in India reported amounts of lead that were in excess of the permissible limits in samples of Magi two-minute noodles (Nestle, Vevey, Switzerland), which is one of the most trusted food brands in India. This finding raised a serious concern about the routine testing of products from the processed food industry [32]. We calibrated the potentiometric response of the DEP chip using standard solutions of cadmium and lead and then applied that calibration to rice and noodle samples. The measured concentrations of all the samples fall within the permissible range and they were further confirmed by testing additionally added known concentrations (Table 2).

### 3.4. On-Site Detection and Validation of Heavy Metals in Drinking Groundwater Samples

**Figure 5:**
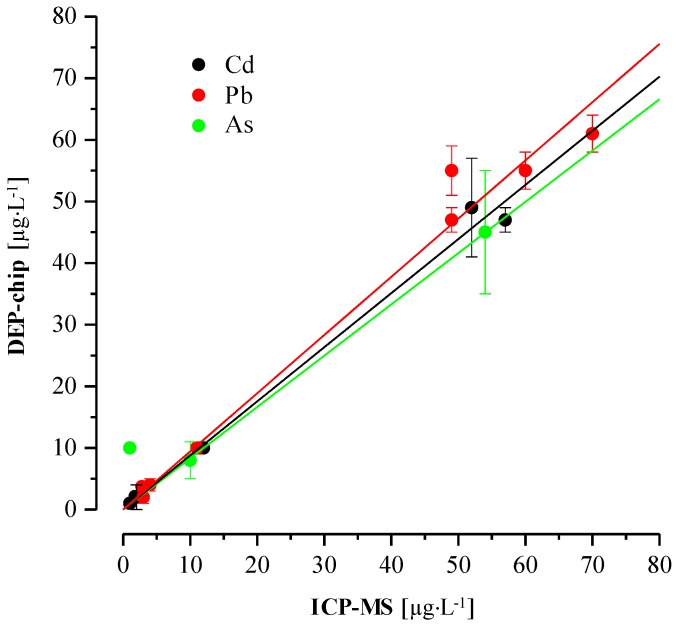
A correlation curve between the conventional (ICP-MS) and “DEP-On-Go” device for sensitive detection of cadmium, lead, and arsenic heavy metals in drinking groundwater samples.

To demonstrate the on-field application for heavy metal ion determination in real drinking groundwater samples, we conducted field experiments to evaluate the on-site rapid detection ability of our DEP-On-Go platform. Some typical heavy metals, including lead, cadmium, and arsenic, are the most alarming contaminants in the groundwater in the northeastern parts of India [33,34]. Several groundwater samples were collected from rural and urban areas in the northeastern parts of India and they were directly measured by our device. As shown in Table 2, we detected high amounts of lead, i.e., near six times above the permissible limit, from the northern parts of India and verified the ability of our device for early warning of heavy metal toxicity in polluted residential areas. In order to validate the DEP chip-based electrochemical detection method, the results were compared with those obtained by inductively-coupled plasma mass spectrometry (ICP-MS). As shown in Table 2, the concentrations of lead, cadmium, and arsenic determined by the DEP chip were very close to those determined using the ICP-MS method, and they showed a linear correlation curve with a correlation coefficient ranging from 0.995 to 0.978 (Figure 5). These proof-of-principle experiments and the comparative study suggest that our DEP chip-based platform has promising possibilities for practical applications. We believe that the “DEP-On-Go” device is highly desirable for the rapid initial screening of environmental samples and is, thus, anticipated as a heavy metal surveillance method in healthcare management for providing the early alarming signs of heavy metal toxicity.

## 4. Discussion

The simultaneous detection of several heavy metals is advantageous over time/cost-consuming manual operations, particularly when real samples, such as drinking groundwater, are polluted with a mixture of several heavy metals. Nevertheless, the signal of the target metal often experiences interference because of the presence and co-deposition of other heavy metals onto the active site of the electrode surface. The interference effect from a mixture of other heavy metal ions (zinc, cadmium, lead, copper) on the detection of each of zinc, cadmium, lead, and copper was investigated using a carbon DEP chip and without the addition of bismuth. The DPV profiles resulting from the simultaneous detection of zinc, cadmium, and lead on the carbon DEP chip can be seen in the supporting materials (Figure S6). A significant loss in the peak current heights of zinc was observed, even in the presence of trace amounts of cadmium and lead (*t* test, ** *p* < 0.01). However, this could be improved by the co-deposition of bismuth. The cross-interference effect of electrochemical co-detection of heavy metals can be seen in the supporting materials (Figure S7): the stripping peak current of zinc in the presence of a mixture of other heavy metals (cadmium, lead, copper) gave rise to a signal loss about 31%. However, there were no changes in the stripping responses of copper, and much less significant change in the responses of cadmium and lead in the presence of other heavy metals. Next, the interference effect from simultaneous additions of other heavy metals (e.g., cadmium, lead, and copper) on the detection of arsenic on gold DEP chips was studied (Figure S8). The peak current height for arsenic was shown to grow in magnitude with increasing additions (10 to 200 μg·L^−1^) and a small loss of the peak current heights in the presence of other heavy metals. No significant interference was observed on the arsenic peak current height at the 10–150 μg·L^−1^ concentrations. However, a small decrease in the peak current height was observed in the presence of a mixture of cadmium and cooper or lead and copper at higher concentrations above 150 μg·L^−1^ (using ANOVA—Analysis of variance, ** *p* < 0.01). These results indicate that the gold DEP chip can be used to detect arsenic for simultaneous monitoring with minimal interference caused by other co-deposited foreign metals.

## 5. Conclusions

A simple and affordable mobile electrochemical detector system for unreached communities is described for the rapid on-site monitoring of toxic heavy metals in environmental samples, including drinking groundwater, house dust, soil, and food samples. The unique combination of portable hardware, including the disposable electrode printed (DEP) chip, a palm-sized potentiostat, and smartphone-supported software for differential pulse voltammetry (DPV) analysis were used as key characteristics of this mobile system. Finally, the system was used to detect multiple heavy metals simultaneously in real samples as an example of field applications with great affordability (less than one US dollar) that are fast (sample-to-answer in less than 5 min), and have high sensitivity (ppb levels and below permissible range). Future work will focus on the extension and utility of this system for other untested and challenging pollutants.

## Figures and Tables

**Table 1:** Detectable efficiency and the found values of heavy metals in environmental samples using the DEP-On-Go system against quality guidelines from several environmental protection organizations.

	Limit of Detection	Drinking Water		Air Dust		Soil	
		PEL ^a^	Sample ^d^	PEL ^b^	Sample ^e^	PEL ^c^	Sample ^f^
	(μg·L^−1^)	(μg·L^−1^)		(μg∙m^−3^)	(μg∙g^−1^)	(μg∙g^−1^)	
**Cd**	2.6	3	1.73	5	0.22	85	0.016
**Pb**	4.0	10	6.5	50	27.4	420	0.24
**As**	5.0	10	2.3	10	0.034	75	0.025
**Hg**	1.5	6	4.1 *	25	27.2 *	840	0.045 *
**Cu**	15.5	2000	14.7	1000	8.14	4300	0.175
**Zn**	14.4	3000	1240	10,000	75.6	7500	1.4

^a^ WHO (World Health Organization) [25]; ^b^ OSHA (Occupational Safety and Health Administration of United States, Department of Labor) [26]; ^c^ US-EPA (United States Environmental Protection Agency) [27]; ^d^ Drinking groundwater sample from central Jaipur city, India; ^e^ Room dust from an educational campus in central Jaipur city, India; ^f^ Soil from a garden area in Nomi city, Japan; * combined values of mercury with iron. PEL: Permissible Exposure Limit.

**Table 2:** A comparison of detecting heavy metals (μg·L^−1^ added) in samples of water (groundwater, tap water), food (rice and noodles), and dust between ICP-MS and “DEP-On-Go” methods.

	Samples	ICP-MS	DEP Chip	Recovery (%)
**Lead**				
1	Noodles (Brand-A, India) 1g/50 mL	2.8	3.7	132
2	Noodles (Brand-B, Japan) 1g/50 mL	1	ND	-
3	Noodles (Brand-B, Japan + 10 μg·L^−1^ added)	11	10 ± 1	91
4	Noodles (Brand-B, Japan + 50 μg·L^−1^ added)	49	55 ± 4	112
5	Groundwater (Bikaner, Rajasthan, India)	70	61 ± 3	87
6	Groundwater (Amarapura, Rajasthan, India)	60	55 ± 3	92
7	Tap water (Nomi, Ishikawa, Japan)	3	2 ± 1	67
8	Tap water (Nomi, Ishikawa, Japan + 1 μg·L^−1^ added)	4	4 ± 1	100
9	Tap water (Nomi, Ishikawa, Japan + 50 μg·L^−1^ added)	49	47 ± 2	96
10	Dust (Jaipur, Rajasthan, India) 1 g/ 50 mL	910	800 ± 63	89
11	Dust (Nomi, Ishikawa, Japan) 1 g/50 mL	520	510 ± 19	98
**Cadmium**				
12	Rice (Indica brand-A, India) 1g/50 mL	1.8	2.1	116
13	Rice (Japonica brand-B, Japan) 1g/50 mL	1	ND	-
14	Rice (Japonica brand-B, Japan + 10 μg·L^−1^ added)	12	<10	-
15	Rice (Japonica brand-B, Japan + 50 μg·L^−1^ added)	52	49 ± 8	94
16	Tap water (Nomi, Ishikawa, Japan)	<1	<1	-
17	Tap water (Nomi, Ishikawa, Japan + 1 μg·L^−1^ added)	2	2 ± 2	100
18	Tap water (Nomi, Ishikawa, Japan + 50 μg·L^−1^ added)	57	47 ± 2	83
**Arsenic**				
19	Tap water (Nomi, Ishikawa, Japan)	<1	<10	-
20	Tap water (Nomi, Ishikawa, Japan + 10 μg·L^−1^ added)	10	8 ± 3	80
21	Tap water (Nomi, Ishikawa, Japan + 50 μg·L^−1^ added)	54	45 ± 10	83

## Supplementary Materials

The following are available online at http://www.mdpi.com/1424-8220/17/1/45/s1, Figure S1: A cyclic voltammogram of the conventional bench-top unit and our hand-held potentiostat in 0.5 M Na_2_SO_4_ solution containing 2.0 mM hexacyanoferrate. Figure S2: Calibration curves for the simultaneous electrochemical detection of zinc, cadmium, lead, and copper using a carbon DEP chip (C-DEP) and arsenic, and mercury using a gold DEP chip (Au-DEP) in standard solutions. The *X*-axis and *Y*-axis represent the concentrations of metals (μg·L^−1^) and calculated peak current heights (μA), respectively. The data are the averages of four to six independent experiments. LoD: Limit of detection. Figure S3: Calibration curves for the simultaneous detection of zinc, cadmium, lead, copper and mercury in real drinking groundwater sample. The *X*-axis and *Y*-axis represent the concentrations of the metals (μg·L^−1^) and the calculated peak current height (μA), respectively. The data are the averages of four to six independent experiments. Figure S4: Calibration curves for the simultaneous detection of zinc, cadmium, lead, copper and mercury in real house dust sample. The *X*-axis and *Y*-axis represent the concentrations of the metals (μg·L^−1^) and the calculated peak current height (μA), respectively. The data are the averages of four to six independent experiments. Figure S5: Calibration curves for the simultaneous detection of zinc, cadmium, lead, copper and mercury in real garden soil sample. The *X*-axis and *Y*-axis represent the concentrations of the metals (μg·L^−1^) and the calculated peak current height (μA), respectively. The data are the averages of four to six independent experiments. Figure S6: Simultaneous detection of heavy metals using a carbon DEP chip. DP voltammograms (a) and the corresponding interference effect (b) of zinc in the presence of cadmium and lead. Figure S7: The cross-interference effect on carbon DEP chip in electrochemical co-detection of heavy metals. Individual heavy metals including zinc, cadmium, lead and copper were measured with and without the successive additions of a mixture of other heavy metals. Figure S8: Simultaneous detection of heavy metals using the gold DEP chip. The DP voltammograms (a) and corresponding interference effect (b) of arsenic in the presence of cadmium and copper (top left) and lead and copper (top right). Table S1: DPV parameters for the detection of different heavy metals on carbon and gold DEP chips. Table S2. Comparative features of ICP-MS and DEP chip methods. Video S1: Real-time video of on-site field operation of “DEP-on-Go” system in the northern parts of India (Jaipur City) for detection of heavy metals in real drinking groundwater sample.
