# Supplementary material for: DEP-On-Go for Simultaneous Sensing of Multiple Heavy Metals Pollutants in Environmental Samples
Source: Sensors (Basel). 2016 Dec 27;17(1):45. doi: 10.3390/s17010045 (PMC5298618; doi:10.3390/s17010045)
Supplement: Supplementary file 1 [file sensors-17-00045-s001.zip › sensors-17-00045-suppl/sensors-17-00045-suppl.pdf]

Supplementary Materials: DEP-On-Go for Simultaneous Sensing of Multiple Heavy Metals Pollutants in Environmental Samples

Madhu Biyani, Radhika Biyani, Tomoko Tsuchihashi, Yuzuru Takamura, Hiromi Ushijima, Eiichi Tamiya and Manish Biyani

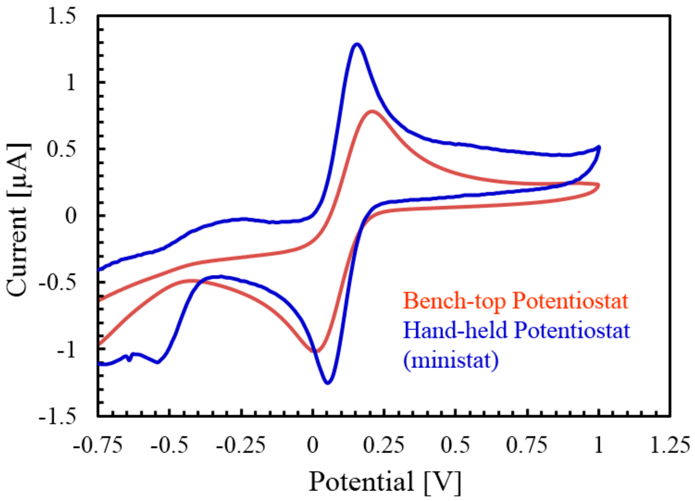

Figure S1. A cyclic voltammogram of the conventional bench-top unit and our hand-held potentiostat in 0.5 M Na_2SO_4 solution containing 2.0 mM hexacyanoferrate.

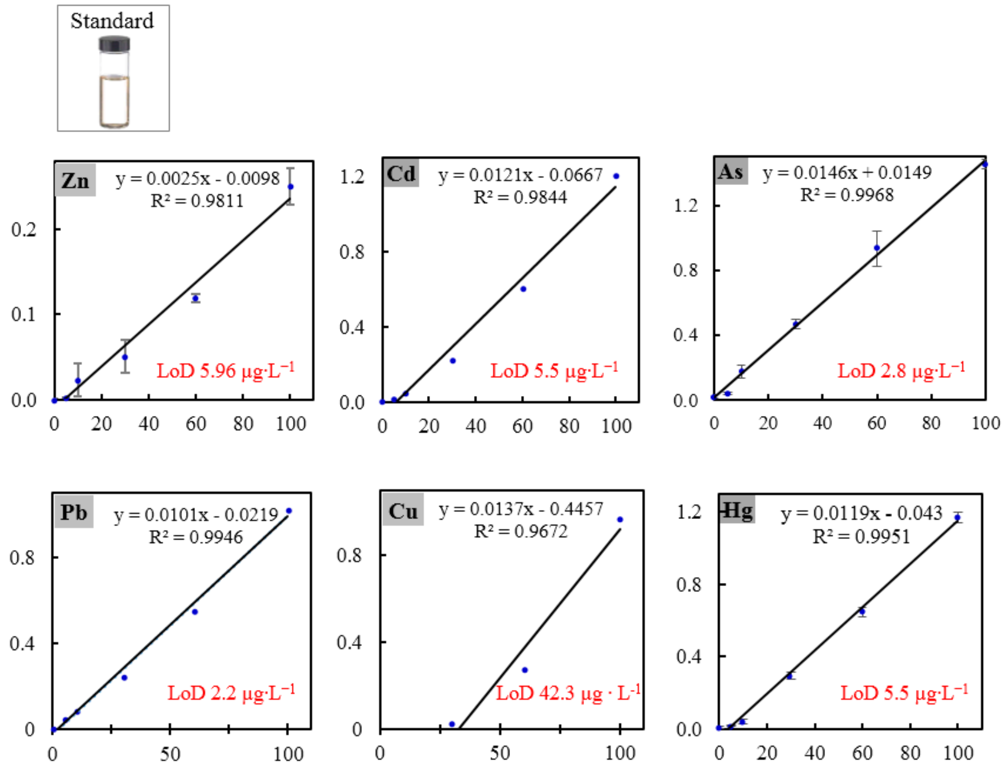

Figure S2. Calibration curves for the simultaneous electrochemical detection of zinc, cadmium, lead, and copper using a carbon DEP chip (C-DEP) and arsenic, and mercury using a gold DEP chip (Au-DEP) in standard solutions. The *X*-axis and *Y*-axis represent the concentrations of metals (μg·L^{-1}) and calculated peak current heights (μA), respectively. The data are the averages of four to six independent experiments. LoD: Limit of detection.

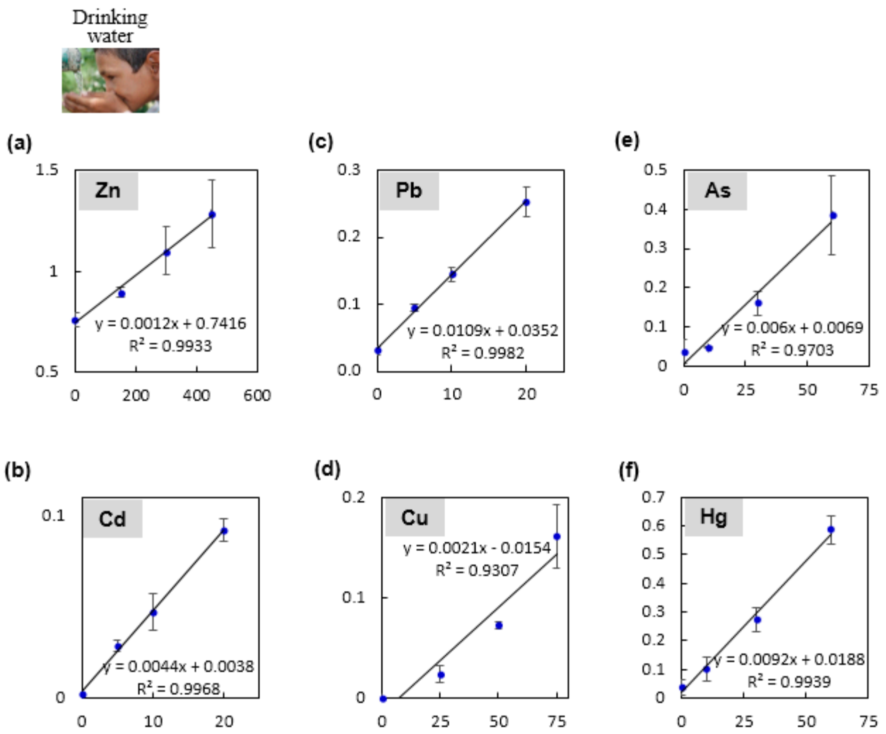

Figure S3. Calibration curves for the simultaneous detection of zinc (**a**); cadmium (**b**); lead (**c**); copper (**d**); arsenic (**e**); and mercury (**f**) in real drinking groundwater sample. The *X*-axis and *Y*-axis represent the concentrations of the metals (μg·L^{-1}) and the calculated peak current height (μA), respectively. The data are the averages of four to six independent experiments.

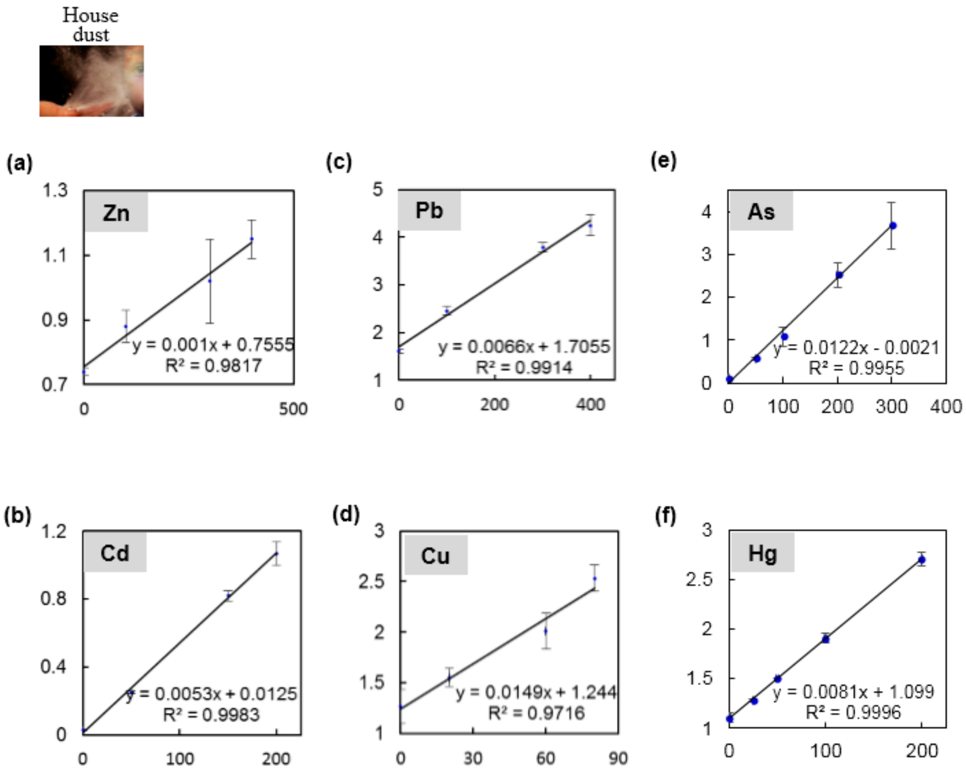

Figure S4. Calibration curves for the simultaneous detection of zinc (**a**); cadmium (**b**); lead (**c**); copper (**d**); arsenic (**e**); and mercury (**f**) in real house dust sample. The *X*-axis and *Y*-axis represent the concentrations of the metals (μg·L^{-1}) and the calculated peak current height (μA), respectively. The data are the averages of four to six independent experiments.

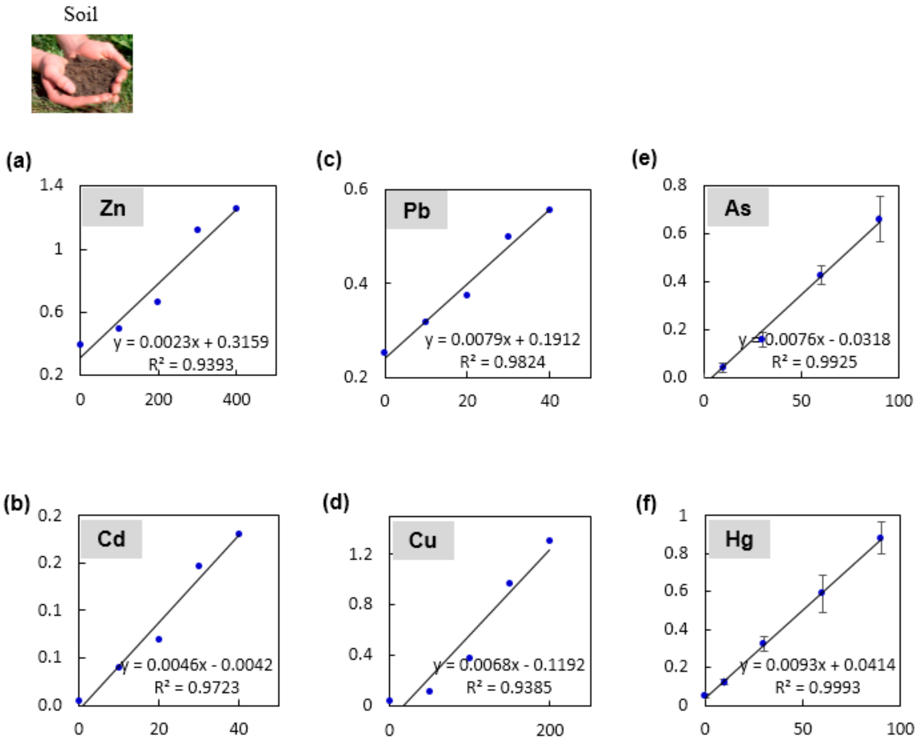

Figure S5. Calibration curves for the simultaneous detection of zinc (**a**); cadmium (**b**); lead (**c**); copper (**d**); arsenic (**e**); and mercury (**f**) in real garden soil sample. The *X*-axis and *Y*-axis represent the concentrations of the metals (μg·L⁻¹) and the calculated peak current height (μA), respectively. The data are the averages of four to six independent experiments.

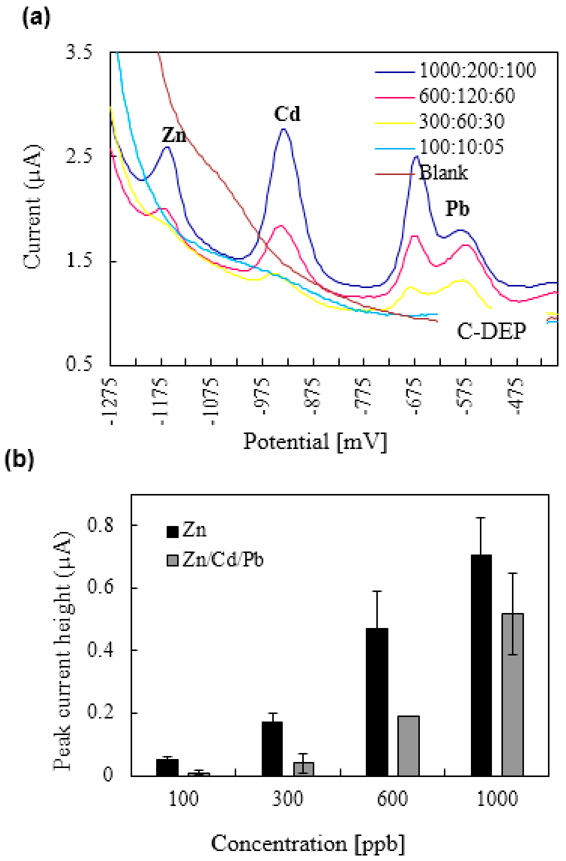

Figure S6. Simultaneous detection of heavy metals using a carbon DEP chip. DP voltammograms (**a**) and the corresponding interference effect (**b**) of zinc in the presence of cadmium and lead.

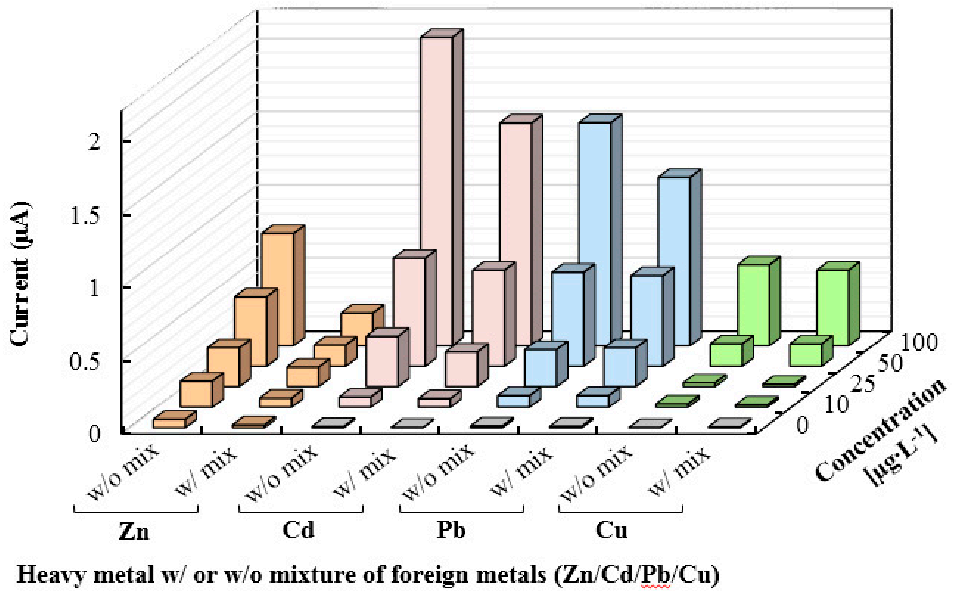

Figure S7. The cross-interference effect on carbon DEP chip in electrochemical co-detection of heavy metals. Individual heavy metals including zinc, cadmium, lead and copper were measured with and without the successive additions of a mixture of other heavy metals.

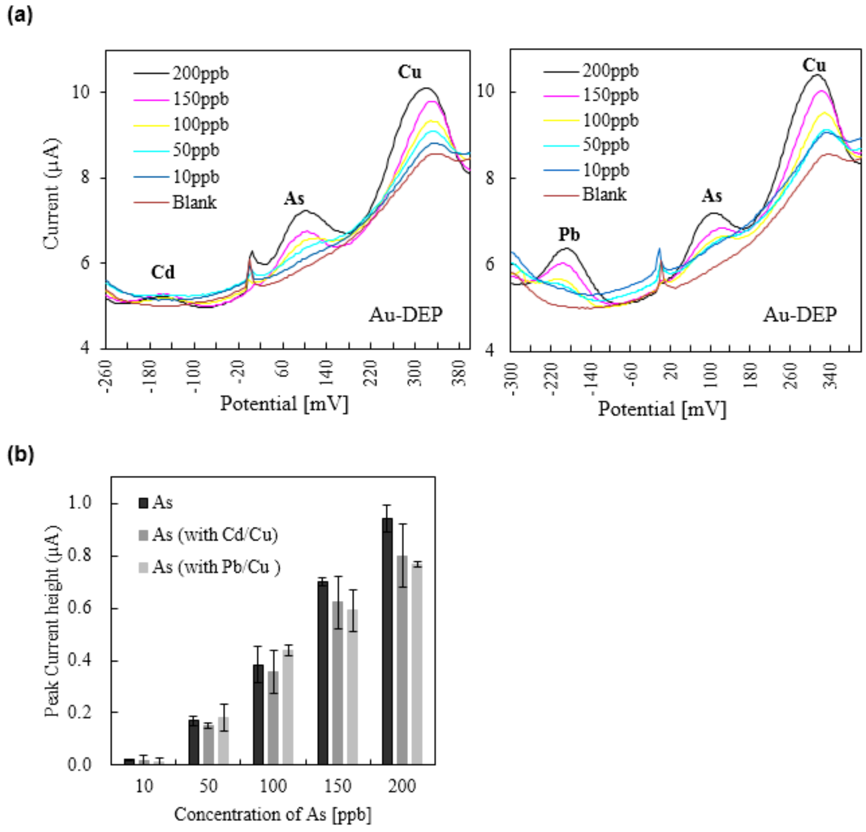

Figure S8. Simultaneous detection of heavy metals using the gold DEP chip. The DP voltammograms (**a**) and corresponding interference effect (**b**) of arsenic in the presence of cadmium and copper (top left) and lead and copper (top right).

Table S1. DPV parameters for the detection of different heavy metals on carbon and gold DEP chips

DPV Parameters	Carbon DEP-Chip					Gold DEP-Chip		
	Zn	Cd	Pb	Cu	Zn/Cd/Pb/Cu	As	Hg	As/Hg
Peak potential (V)	−1.423	−1.178	−0.889	−0.321	−1.428/−1.109/−0.887/−0.3	0.092	0.45	0.06/0.42
**Potential**								
Begining potential (mV)	−1500	−1300	−1300	−490	−1500	−140	300	−100
End potential (mV)	−1200	−900	−700	−130	250	165	600	500
Step amplitude (mV)	4	4	4	4	4	10	10	10
Pulse amplitude (mV)	50	50	50	50	50	50	50	50
**Time**								
Pulse period (ms)	200	200	200	200	200	200	100	100
Pulse width (ms)	50	50	50	50	50	50	40	40
Sampling width (ms)	16	16	16	16	16	16	2	2
Scan rate mV/s	20	20	20	20	20	100	100	100
**Deposition Conditions**								
E1 (mV)	−1600	−1400	−1400	−1400	−1600	−400	200	−250
T1 (s)	300	300	300	300	300	120	120	120
**Range**								
Fixed	1	1	1	1	1	1	1	1

Table S2. Comparative features of ICP-MS and DEP chip methods.

	ICP-MS	Our (DEP-On-Go) System
Set-up cost	$179,000–448,000	$2000 or less
Running cost per sample	$20	$1–2
Sample analysis time	~5 min	Few minutes (1–5 min)
Analytical procedure	Complex	Easy
Portability	No	Yes
Contamination	High	No/Less
Sample preparation	Multi-step	Simple
Sample volume required	mL	μL
Detection limit	extremely sensitive (ppt)	sensitive (sub-ppb)
